# Orthodontically induced external apical root resorption considerations of root-filled teeth vs vital pulp teeth: a systematic review and meta-analysis

**DOI:** 10.1186/s12903-023-02982-4

**Published:** 2023-04-25

**Authors:** Danning Zhao, Kun Xue, Jiayuan Meng, Meijing Hu, Fei Bi, Xuelian Tan

**Affiliations:** 1 West China School of Stomatology, Sichuan University, Chengdu, China; 2 Department of Epidemiology and Health Statistics, West China School of Public Health and West China Fourth Hospital, Sichuan University, Chengdu, China; 3 Department of Orthodontics, State Key Laboratory of Oral Diseases, National Clinical Center for Oral Diseases, West China School of Stomatology, Sichuan University, Chengdu, China; 4 Department of Cariology and Endodontics, State Key Laboratory of Oral Diseases, National Clinical Center for Oral Diseases, West China School of Stomatology, Sichuan University, No. 14, Section 3, South Renmin Road, Chengdu, 610041 China

**Keywords:** Root-filled teeth, Endodontic treatment, Orthodontic treatment, Orthodontic root resorption

## Abstract

**Introduction:**

The purpose of this systematic review was to research the difference between root-filled teeth (RFT) and vital pulp teeth (VPT) in orthodontically induced external apical root resorption (EARR) and to offer suggestions for clinicians on therapeutic sequence and timing when considering combined treatment of endodontic and orthodontic.

**Materials and methods:**

An electronic search of published studies was conducted before November 2022 in PubMed, Web of Science and other databases. Eligibility criteria were based on the Population, Intervention, Comparison, Outcome, and Study design (PICOS) framework. RevMan 5.3 software was used for statistical analysis. Single-factor meta-regression analysis was used to explore the sources of literature heterogeneity, and a random effects model was used for analysis.

**Results:**

This meta-analysis comprised 8 studies with 10 sets of data. As there was significant heterogeneity among the studies, we employed a random effects model. The funnel plot of the random effects model exhibited a symmetrical distribution, indicating no publication bias among the included studies. The EARR rate of RFT was significantly lower than that of VPT.

**Conclusions:**

In the context of concurrent endodontic and orthodontic treatment, priority should be given to endodontic therapy, as it serves as the foundation for subsequent orthodontic procedures. The optimal timing for orthodontic tooth movement post-root canal therapy is contingent upon factors such as the extent of periapical lesion resolution and the degree of dental trauma sustained. A comprehensive clinical assessment is essential in guiding the selection of the most suitable approach for achieving optimal treatment outcomes.

## Introduction

Tooth root resorption is a complex and unpredictable pathological process that relates to cementum, root dentin or apex. Resorption can even cause irreversible loss of tooth structure. Orthodontically induced external apical root resorption (EARR) is a common and deleterious adverse consequence of inflammation-driven tooth movement [1]. Histological studies have shown that EARR occurs in 90% of teeth involved in the orthodontic movement [2]. It has been reported that more than 80% of patients undergoing orthodontic treatment had root resorption of more than 1 mm, and one-third had root resorption of more than 3 mm [3]. According to a new study, the incidence of severe root resorption after orthodontic treatment was 14.8% [4]. Root resorption is considered a particularly important sequela of orthodontic treatment because it can impair the stability of the treatment outcomes and the longevity of the tooth [5].

Root resorption in both physiologic and pathologic instances involves a coordinated interaction among osteoblasts and osteoclasts as well as odontoblasts and odontoclasts that are regulated [6]. Under a stimulus, with a local increase of cytokines, T-cells are activated and express RANKL, and subsequently, differentiation and activation of pre-odontontoclasts occur. Odontoblasts and fibroblasts interact with bioactive neuropeptides. Cytokines, interleukin-β (IL-β) and IL-6, prostaglandin E2, tumour necrosis factor α (TNF-α), and hormones induced by the debilitated periodontal ligament (PDL) stimulate the expression of RANKL by fibroblasts and play a part through their vasoactive, chemotactic, and cellular effects [7]. Those events consequently led to the recruitment of active odontoclasts, which promotes the beginning of the root resorption process.

Influencing factors of EARR include patient age, duration of treatment, the magnitude of orthodontic force, and type of orthodontic devices [8–10]. Published studies have suggested that EARR is associated with previous endodontic treatment and the status of the pulp [2, 11–19]. Pulp reactions cause apical resorption and remodeling during orthodontic movement, so different magnitudes of EARR might occur in root-filled teeth (RFT) and vital pulp teeth (VPT) [20]. Although the scientific reports on the EARR of RFT are few, much debate about its response to orthodontic forces keeps springing up. Khan and Kumar considered that the risk of EARR is higher in endodontically treated teeth in a study of 30 patients [12]. However, the latest research concluded contrarily that RFT presented significantly less EARR than VPT [2, 13–15, 21, 22]. Yoshpe even suggested that endodontic procedures may be effective to treat or prevent external root resorption during orthodontic treatment [16]. Different from above the two views, Llamas-Carreras et al*.* found that there was no significant difference in the degree of EARR between RFT and VPT [17]. Bellini-Pereira concluded that treatment-related factors such as the type of mechanics applied and treatment duration might have a minor influence on EARR [18]. As for the cause of the controversy, Alqerban et al. found that the difference in EARR between RFT and VPT correlated with the quality of endodontic treatment [19]. Souza et al. considered that Ca(OH)2-based materials had a favourable effect on periapical tissue healing of EARR in endodontically treated dogs’ teeth [23]. This may be one reason why RFT has a lower EARR rate than VPT, but no human studies have shown this.

Orthodontically treated teeth sometimes need endodontic treatments, and endodontically treated teeth may also need orthodontic treatments, so this can be a common multidisciplinary problem. However, the possibility of root resorption after orthodontic movement remains controversial [24]. When considering the possibility of EARR, it is crucial to address the following queries: 1) What is the priority treatment when both orthodontic and endodontic procedures are necessary? 2) What is the optimal timing of orthodontic tooth movement after root canal therapy? 3) Should the interval between endodontic and orthodontic treatment be determined by the severity of the periapical lesion? The present study endeavors to determine if there exists a discrepancy in EARR incidence between RFT and VPT following an orthodontic intervention. We aim to conduct a systematic review and meta-analysis of available data to provide qualitative and quantitative insights. Additionally, we will discuss the contributing factors for any observed differences to offer practical recommendations for determining the optimal sequence of endodontic and orthodontic treatments, thus minimizing the risk of EARR in clinical practice.

## Materials and methods

### Protocol and registration

This meta-analysis was conducted according to the *Preferred Reporting Items for Systematic Reviews and Meta-Analyses (PRISMA)* statement [25]. The protocol was registered in PROSPERO with registration number CRD 42021278290.

### Eligibility criteria

Eligibility criteria were based on the Population, Intervention, Comparison, Outcome, and Study design (PICOS) framework:

- Population: Patients of any age after endodontic and orthodontic treatment were included.
- Intervention: Teeth that have undergone endodontic and orthodontic treatment were the studied objects.
- Comparison: Contralateral teeth with vital pulp that underwent orthodontic treatment were used for comparison.
- Outcome: Comparison of orthodontic root resorption was the outcome of interest.
- Study design: The article type was limited to clinical trials. Prospective, retrospective, and cross-sectional studies were reviewed.

### Information sources and searches

An electronic search was conducted for studies published up to November 2022 in the PubMed, Scopus, MEDLINE (Ovid), Web of Science, and Cochrane Library databases. There were no restrictions on publication year, language or status. A search was performed using the following keywords: (“endodontic” OR “root canal”) AND (“root resorption”) AND (“orthodontic”). These search keywords were originally created for PubMed and modified appropriately for the other databases.

### Study selection

Inclusion criteria included the following:

1. Clinical study
2. Sample size given
3. Imaging techniques were used to assess root resorption outcomes
4. Raw data on root resorption before and after orthodontic treatment

Exclusion criteria included the following:

1. Studies conducted using primary teeth or animal models
2. Sample with a particular disease (i.e., diabetes or periodontal disease)
3. Raw data measures were not standardized

Titles and abstracts were screened according to inclusion and exclusion criteria. For potentially useful studies, the same criteria were used for full-text screening. 2 reviewers made separate research selections and extracted data independently according to the predesigned items. A third reviewer was involved when there was a disagreement. Cohen's kappa was used to evaluate the consistency of research qualifications and data extraction among reviewers.

### Data extraction and data items

The following data were extracted from each included study: study characteristics (author, publication year, study design type, the quartile and impact factor of journal), sample characteristics (sample size, patient age, and gender), treatment characteristics (teeth type, treatment type, treatment time and order), and result characteristics (imaging evaluation method and EARR in millimeters).

### Assessment of risk of bias

The risk of bias was assessed using RevMan 5.3 software (Review Manager, Cochrane Collaboration, Copenhagen, Denmark). Since none of the included studies were randomly assigned to the intervention control group, the Risk Of Bias In Non-randomized Studies of Interventions tool (Cochrane Bias Methods Group, Odense, Denmark) was used to assess the estimated risk of bias for the relative effectiveness of the intervention [26]. 2 reviewers evaluated the bias risk of the article from the following seven aspects: random sequence generation, allocation concealment (selection bias), blinding of participants and personnel (performance bias), blinding of outcome assessment (detection bias), incomplete outcome data (attrition bias), selective reporting (reporting bias), and other bias (absence of description regarding tooth location or the amount of remaining coronal structure). The risk for each criterion was reported as low, high or unclear.

### Assessment of publication bias

Publication bias was assessed by visual funnel plot asymmetry. When a funnel plot made it difficult to judge whether publication bias existed subjectively, Egger’s linear regression quantitative test was used.

### Assessment of certainty in the evidence

The quality of evidence included in the study was evaluated, and the quality level of relevant evidence was finally determined [27, 28]. Cohen's kappa was used to evaluate the consistency of quality evaluation by reviewers.

### Synthesis of results

The Excel data were imported into RevMan 5.3 software in the ".xls" format using the meta-analysis module for statistical analysis. A mixed effects model was selected to test the main effect through a heterogeneity test. Single-factor meta-regression analysis was used to explore the sources of heterogeneity in the literature. The Q test and I^2^ statistic were used to test interstudy heterogeneity. When I^2^ < 50% and *p* > 0.1, the fixed effects model was selected for the analysis because the heterogeneity among studies was small. When I^2^ ≥ 50% and *p* ≤ 0.1, the heterogeneity between studies was considered to be large, and the random effects model was selected for the analysis. The differences in EARR and different intervention characteristics were expressed by the standard mean difference (SMD) and 95% confidence interval (CI). The heterogeneity test level was set as α = 0.1, and the remaining test levels were set as α = 0.05.

### Sensitivity analysis

In addition, the effect size of removing individual studies was analyzed to evaluate the robustness and reliability of the combined results.

## Results

### Study selection

**Fig. 1:**
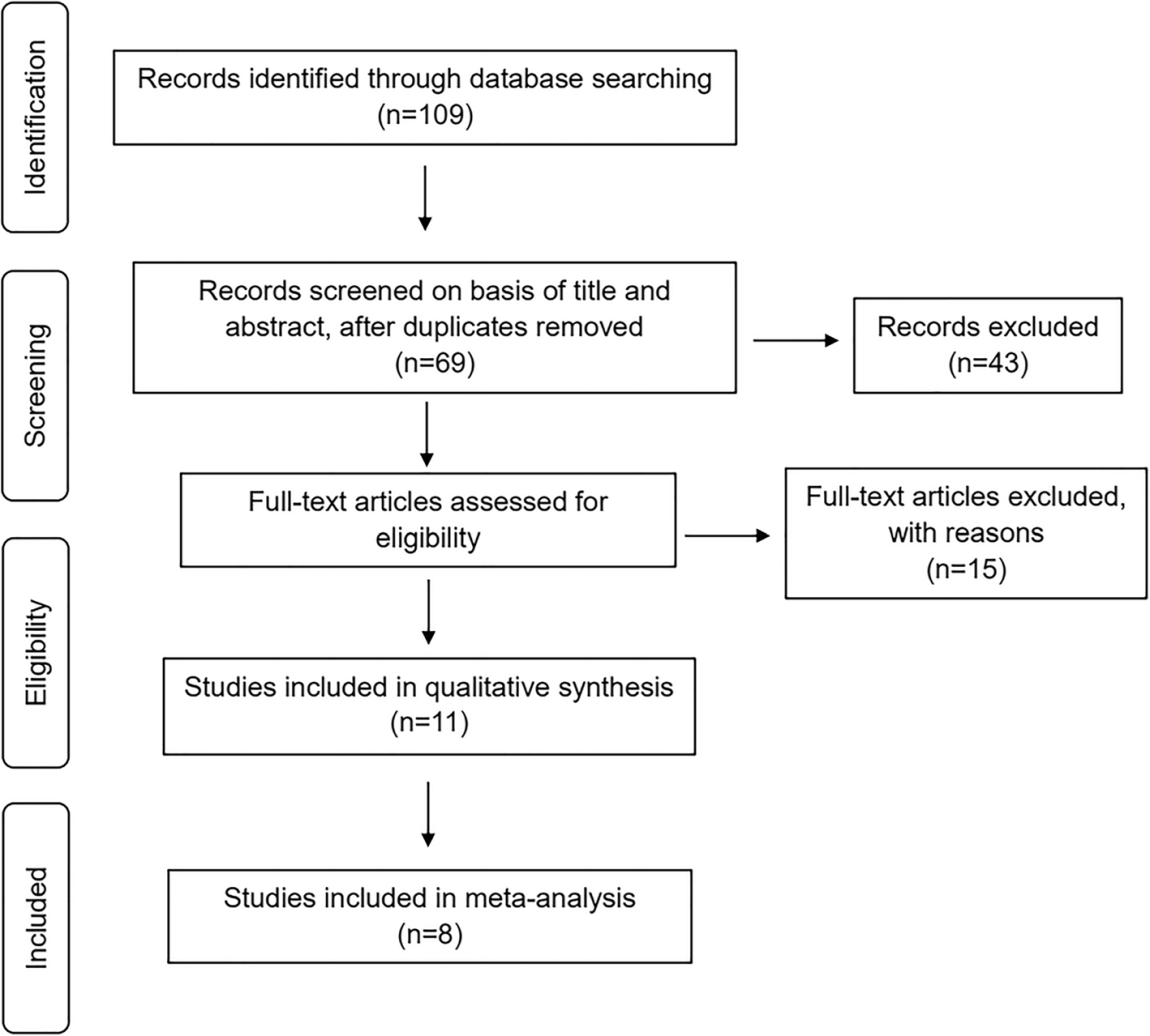
A flow diagram of the study identification and selection process

Initially, the search yielded 109 records (Fig. 1). After removing duplicates, a total of 69 studies were screened by title and abstract. Subsequently, 43 studies were deemed irrelevant to the current review, leaving 26 studies for full-text review. Two independent reviewers assessed the studies, resulting in the exclusion of 15 studies due to inadequate methodological and outcome indicators. Eventually, 11 studies were deemed suitable for inclusion in this meta-analysis. Upon further evaluation, two studies lacked raw data on root resorption, while one study only provided data on root resorption volume but not on root resorption length, leading to their exclusion. The selected studies were either prospective or retrospective controlled clinical trials that satisfied predefined inclusion criteria. Internal auditor consistency was assessed by Cohen's kappa.

### Study characteristics

The characteristics of the included studies are presented in Table 1. Among these, four studies reported that the EARR of VPT was significantly higher than that of RFT [2, 13, 14, 29], and four reported that the EARR of VPT was similar to that of RFT [30–33]. In all studies, root canal therapy preceded orthodontic treatment, and the time interval between root canal therapy and orthodontic treatment was unknown.

**Table 1:** A summary of the descriptive characteristics of the included studies

Authors	The quartil and the journal impact factor	Study	Sample size(number of teeth)	Age in year (average)	Gender and number of patients	Teeth type	Treatment type	Treatment time (average)	Treatment order	Interval between treatments	Evaluation	Root resorption
Spurrier et al., 1990 [29]	Q3, 1.96	Retrospective	43	14	43(21 M, 22F)	Maxillary incisors	Multiband/bracket	25 months	Root canal therapy first	NR ^c^	Periapical	RFT ^b^ is less
Esteves et al., 2007 [30]	Q2, 3.118	Retrospective	16	NR	16(NR)	Maxillary Incisors	Brackets	> 20 months	Root canal therapy first	> 1 year	Periapical	No significant difference
Llamas-Carreras et al., 2012 [32]	Q4, 1.596	Prospective	38	30.7 ± 10.2	38(14 M, 24F)	Maxillary incisors	Multiband/bracket	24.0 ± 12.0 months	Root canal therapy first	NR	Digital panorama	No significant difference
Castro et al., 2015 [31]	Q3, 1.549	Prospective	20	12.8 ± 1.8	6(2 M, 4F)	Posterior teeth	Straight-wire technique	22 months	Root canal therapy first	NR	CBCT ^a^	No significant difference
Lee and Lee, 2016 [13]	Q3, 1.96	Prospective	35	25.23 ± 4.92	35(8 M, 27F)	NR	Multiple	26.58 ± 8.58 months	Root canal therapy first	NR	Digital panorama	RFT is less
Wang et al., 2017 [33]	NR	Retrospective	60	12 ~ 38	60(30 M, 30F)	NR	Straight-wire technique	1 ~ 1.5 years	Root canal therapy first	NR	Digital panorama	No significant difference
Khan et al., 2018 [14]	NR	Retrospective	30	26.37 ± 2.4	30(17 M, 13F)	NR	NR	3.17 ± 1.09 years	Root canal therapy first	NR	Orthopantomogram	RFT is less
Kurnaz et al., 2021 [2]	Q4, 1.573	Retrospective	35	18.16 ± 3.79	35(17 M, 18F)	Mandibular molars	Brackets	1.53 ± 0.64 years	Root canal therapy first	> 1 year	Digital panoramic radiograph	RFT is less
Kurnaz et al., 2021b [2]	Q4, 1.573	Retrospective	34	17.72 ± 2.78	34(15 M, 19F)	Mandibular molars	Brackets	2.07 ± 0.72 years	Root canal therapy first	> 1 year	Digital panoramic radiograph	RFT is less

^a^*CBCT* Cone-beam computed tomography

^b^*RFT* Root-filled teeth

^c^*NR* Not report

### Assessment of risk of bias

**Fig. 2:**
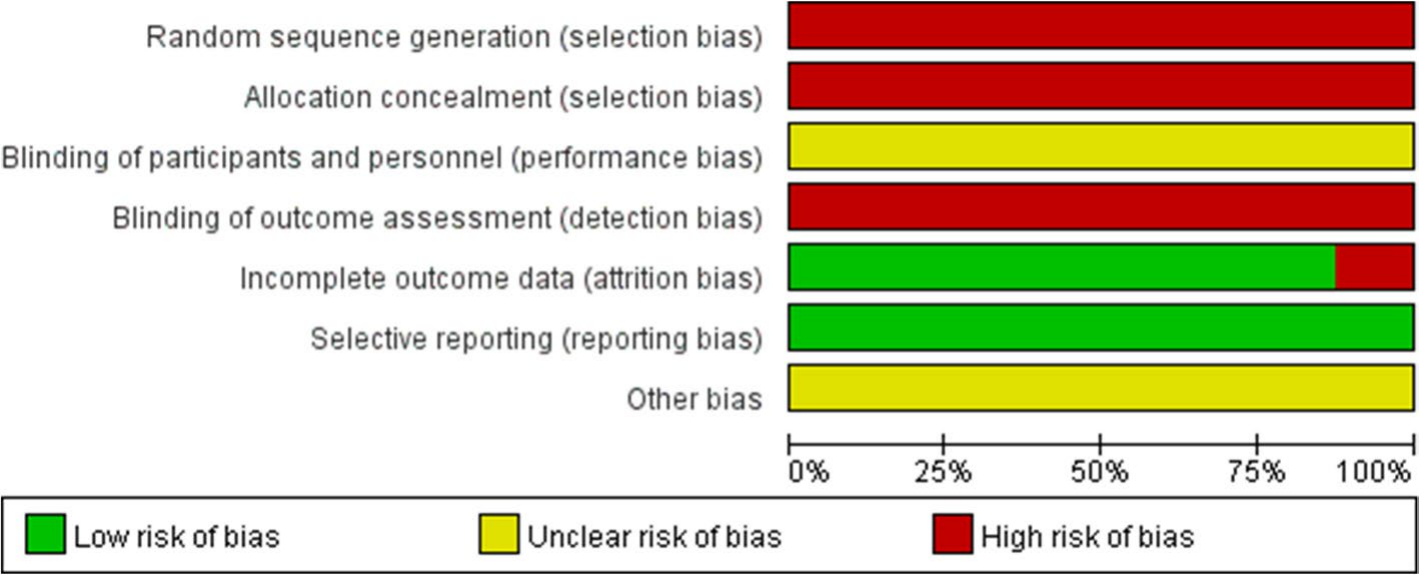
Risk of bias assessment of the included studies. Deviation risk was assessed using deviation risk assessment tools from nonrandomized intervention studies. Bias was evaluated from five aspects: selection bias, performance bias, detection bias, attrition bias and reporting bias. Red indicates high risk, yellow indicates unclear risk, and green indicates low risk in the assessment results. This chart is derived from a review of the authors' selection of risk of bias items for each included study

The included studies were controlled clinical trials, so deviation risk assessment tools in nonrandomized intervention studies were used to assess deviation risk (Fig. 2). Failure to use random sequence generation, allocation concealment, and blinding could result in selection bias. EARR measurements were biased due to the use of different types of radiography. All studies reported predetermined outcome measures. None of the included studies accounted for all possible confounding factors.

### Assessment of publication bias

The funnel plot exhibited a symmetrical distribution, indicating that there was no evidence of publication bias of the data in the source studies. Egger linear regression quantitative test was adopted, *p* > 0.05, and 95%CI include 0, indicating that there is no publication bias of the research data (Table 2).

**Table 2:** Egger linear regression publication bias test

Variable	Estimate	SE	*T* value	*P* value	95%CI
orthodontic root absorption	-1.023	0.883	-1.16	0.271	(-2.97, 0.921)

### Assessment of certainty in the evidence

The grade of evidence was evaluated (Table 3). Uncertainty was not adequately controlled, so the risk of bias was serious. There was no indirect comparison, population difference, or other issues in the included literature, so indirectness was not serious. The number of observed cases was small, so the imprecision was serious. The initial grade for observational tests was low. The final certainty was very low.

**Table 3:** GRADE Working Group grades of evidence

Assessment of certainty					
Risk of bias	Inconsistence	Indirectness	Imprecision	Publication bias	Certainty
Serious	Very serious	Not serious	Serious	Undetected	⊕ 〇〇〇
					Very low

### Synthesis of results

**Fig. 3:**
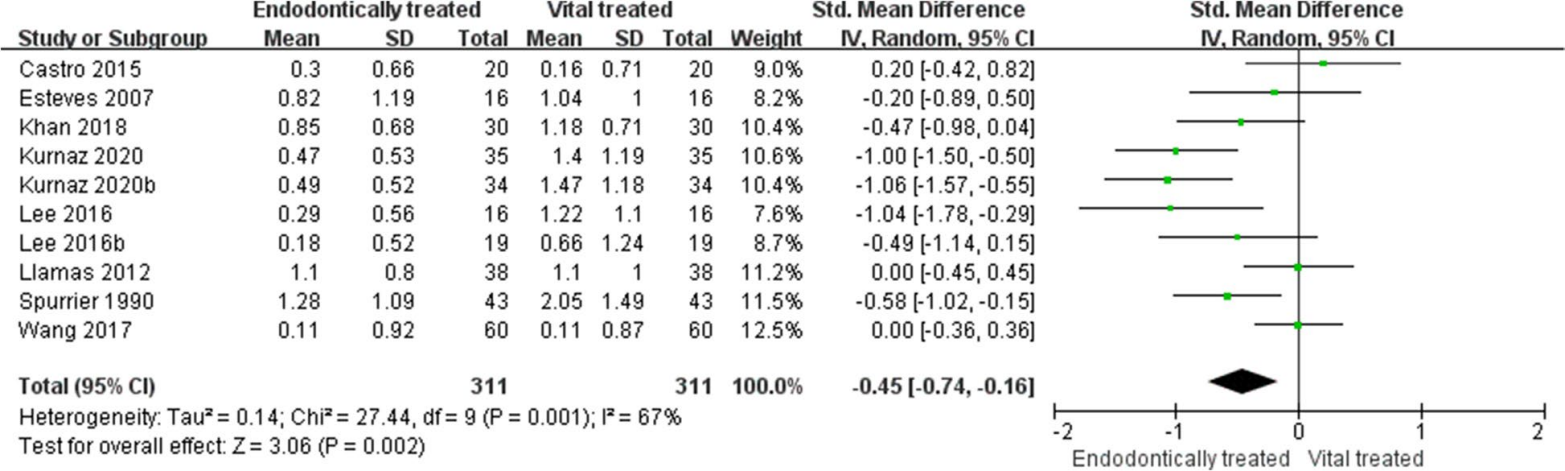
Forest plot of orthodontic root resorption after endodontic treatment and vital pulp teeth. The results of the literature heterogeneity test showed large heterogeneity between studies, so a random effects model was used for analysis. The results of the main effect test showed that orthodontic root resorption of root-filled teeth was lower than that of vital pulp teeth. SD, standard deviation; IV, information value; 95% CI, 95% confidence interval

Eight studies researched the EARR of RFT and VPT. The results of the literature heterogeneity test showed large heterogeneity among studies (I^2^ = 76%, *p* = 0.001), therefore, a random effects model was used for analysis. The results of the main effect test showed an SMD = -0.45, 95% CI (-0.74, -0.16),* p* = 0.002, indicating that the EARR of RFT was significantly lower than that of VPT (Fig. 3).

**Fig. 4:**
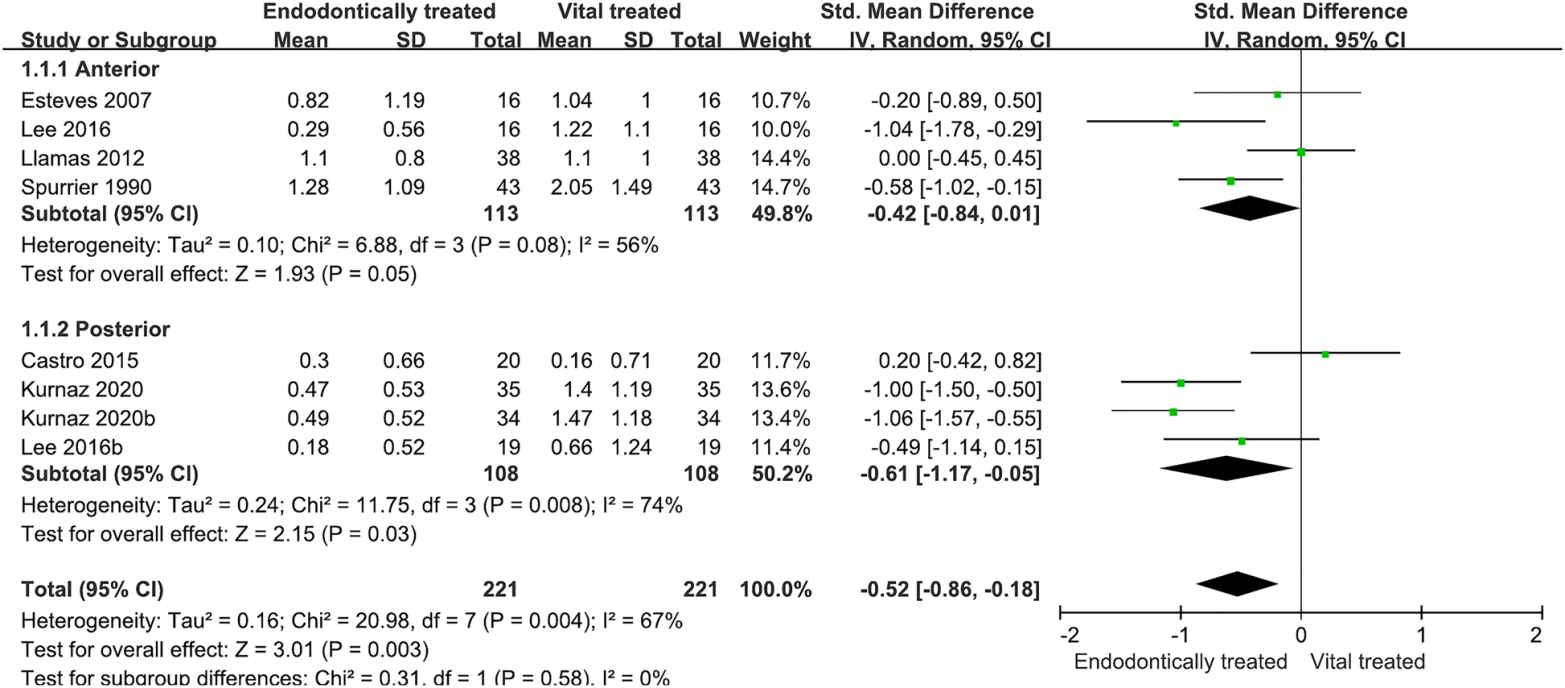
Results of dental subgroup analysis. The teeth were divided into two subgroups by anterior teeth and posterior teeth. Only in the posterior teeth group was the difference between root-filled teeth and vital pulp teeth in terms of the degree of orthodontic root resorption statistically significant. SD, standard deviation; IV, information value; 95% CI, 95% confidence interval

The teeth were divided into 2 subgroups by anterior teeth and posterior teeth. In the anterior group, the SMD = -0.42, 95% CI (-0.84, 0.01), *p* = 0.05, so it could not be considered that the difference between RFT and VPT in terms of EARR was statistically significant. In the posterior group, the SMD = -0.61, 95% CI (-1.17, -0.05), *p* = 0.03, so it could be considered that the EARR of RFT was significantly lower than that of VPT (Fig. 4).

The standardized effect size was used as the Y variable, and the study characteristics such as treatment duration were coded and then set as the X variable for single-factor meta-regression analysis. The age and treatment duration of the subjects could not be considered sources of interstudy heterogeneity.

### Sensitivity analysis

Ten sets of data are covered in 8 studies, analysis of the included literature showed SMD = -0.45, 95% CI (-0.74, -0.16), *p* = 0.002. When 1 study was excluded, the result showed SMD = -0.52 ~ -0.38. There was no statistically significant change in the total effect after removing a certain group of data, and the result was relatively robust.

## Discussion

### Summary of the evidence

The results indicated that EARR was significantly less in RFT than in VPT. This conclusion supported the views obtained in the previous systematic review and meta-analysis [21, 22]. In the subgroup analysis of tooth type, only in the posterior teeth group, there was a statistically significant difference between RFT and VPT in terms of EARR. Root resorption after orthodontic treatment is considered superficial resorption or temporary inflammatory resorption [34]. Gonzales et al. indicated that light orthodontic forces can reduce the risk of inflammatory root resorption in a rat model, but the mechanism remains unclear. Masato Kaku et al. reported that injured and stretched pulp cells express inflammatory cytokines, macrophage colony-stimulating factor (M-CSF), and receptor activator of NF-κB ligand (RANKL), thereby initiating odontoclastic activity. However, these factors would not be secreted without the pulp, and these pulp tissue alterations might explain the increased EARR in VPT [35]. The causes of EARR differences between RFT and VPT need to be further studied.

Regarding the influencing factors of EARR, the age of patients and the time of orthodontic treatment were not found to be correlated with EARR in this study. Many studies have shown that the degree of EARR is related to tooth position, but there is no consensus on the order of how much the EARR of each tooth position is. The majority of studies showed that the EARR of the anterior teeth is greater than that of the posterior teeth [10, 36]. In this study, we also found that the difference in EARR between RFT and VPT was different in the anterior and posterior. In the anterior group, there was no statistically significant difference in EARR between RFT and VPT; while in the posterior group, the EARR of RFT was significantly lower than that of VPT. Whether traumas are risk factors for EARR are debatable. Castro et al. considered that EARR occurred more frequently in maxillary incisors due to trauma, which is not always remembered or mentioned by patients or parents [31]. Li and Fang reported that the incidence and severity of EARR with clear aligners were lower than those with fixed appliances [37, 38].

There are still no clear conclusions about the timing of orthodontic treatment after root canal treatment. Lee and Castro et al. confirmed that EARR was significantly greater in RFT with periapical pathosis before orthodontic treatment from a controlled clinical study [23, 39], which indicated that the EARR of RFT is related to the healing degree of periapical lesions after root canal therapy. Consolaro et al. suggested that force can be applied within a few days after endodontic treatment if the treated tooth has no periapical lesions or inflammatory periapical lesions [40]. Al-Tammami et al. suggested that if there is significant periapical radiolucency, orthodontic treatment should be delayed 6 months until radiographic evidence of healing appears [41].

According to Consolaro et al. and Pustułka et al., teeth with mild trauma and an intact periodontal membrane may require a waiting period of 3–4 months to allow for the restoration of normal periodontal tissue and structure before orthodontic movement is initiated. This waiting period is necessary to prevent potentially adverse effects on the healing process and minimize the risk of further damage to the traumatized tooth [11, 40]. Moderately traumatized teeth require a waiting period of at least 1 year until periapical radiography and/or tomography shows normalized results. In cases where the trauma is severe, such as a root fracture, the waiting period should be extended to 2 years or more. Ariffin et al. conducted research that suggested no elevated risk of tooth resorption associated with mild to moderate trauma if orthodontic treatment was initiated 4–5 months after the injury. Additionally, there was no evidence of inflammatory resorption observed in their study [42]. For the sake of the maximum benefit to the patient, clinical and X-ray monitoring should be performed 6 months after active mobile orthodontics are applied [39]. Therefore, for the teeth undergoing root canal therapy due to trauma, the orthodontic treatment plan should be considered comprehensively according to the degree of trauma and imaging findings.

### Limitations

In the absence of original and high-quality randomized controlled trials, the results of the current study should be interpreted with caution. At present, there are many controversies about the risk factors for orthodontic root resorption, which may be due to the different measurement methods (CBCT results are considered more reliable) or the limitations in the study sample size [10]. Panoramic radiography is a classical technique that has been used to assess EARR, the studies included in this review are mostly based on panoramic imaging, but which has been proven to have low reproducibility due to inclination changes during orthodontic treatment, especially in anterior teeth, so this is also one of the limitations of this study. In addition, the samples may include other hidden confounders, such as previously traumatized teeth. In the future, higher quality clinical data are needed, controlling for confounding factors as much as possible and evaluation with CBCT.

### Clinical suggestions

Clinicians must carefully consider the safety of orthodontic movement following the essential endodontic treatment. In cases where both endodontic and orthodontic therapies are required, we recommend that root canal therapy should take priority. In the event of severe EARR during orthodontic treatment, force application should be discontinued, and an endodontic consultation should be sought to determine the need for root canal therapy, particularly in relation to anterior teeth.

Orthodontic forces can be applied within a few days after root canal therapy if there is no sign of periapical lesions and traumatic source of pathogenesis. If there is a large periapical radiolucency, orthodontic treatment should be delayed by 6 months, and apical radiolucency reduction should be determined before treatment.

Teeth that have been treated with root canal therapy after trauma need to be considered carefully. For teeth with mild trauma, 3–4 months after root canal treatment must pass before orthodontic treatment. For teeth with moderate damage, a year must pass until the periapical X-rays show normal. In more severe cases, 2 years or more must pass. According to the degree of tooth injury, follow-up should be carried out for 3 to 12 months as recommended by the clinician.

Aidos et al. have shown that orthodontics movements promote external resorption and endodontic treatment is mandatory in severe EARR [6]. Bioceramic-based endodontic sealers (calcium-silicate and calcium-phosphate-based) present high bio-compatibility and bioactivity with cicatrization properties and new hard tissue formation. The initial dressing of calcium hydroxide followed by obturation with bioceramic sealers may be considered an alternative treatment modality for several types of root resorption, including EARR without trauma [6].

## Conclusions

- RFT shows relatively lower EARR after orthodontic treatment, and orthodontic movement after root canal treatment can be considered a relatively safe treatment by clinicians.
- Endodontic treatment should be carried out first when both endodontic and orthodontic treatment is needed. The timing of orthodontic treatment should be determined according to the healing degree of periapical lesions and the severity of trauma if the teeth were injured, and it is best to start when the periapical X-rays show normal.
- Studies referring to questions such as the optimal timing of endodontic-orthodontic treatment considering the effect of different periapical infection states and the size of apical radiolucency on orthodontic EARR should be conducted in the future.

## Data Availability

All data generated or analysed during this study are included in this published article.
